# A Novel Radiation Method for Preparing MnO_2_/BC Monolith Hybrids with Outstanding Supercapacitance Performance

**DOI:** 10.3390/nano8070533

**Published:** 2018-07-14

**Authors:** Fan Yang, Xichuan Liu, Rui Mi, Lei Yuan, Xi Yang, Minglong Zhong, Zhibing Fu, Chaoyang Wang, Yongjian Tang

**Affiliations:** 1 Science and Technology on Plasma Physics Laboratory, Research Centre of Laser Fusion, China Academy of Engineering Physics, Mianyang 621900, China; yangfanxiu@163.com (F.Y.); liuxichuan098@163.com (X.L.);mirui_00@163.com; yuanlei0211@163.com (L.Y.); xingxingysx@163.com (X.Y.); 13110200008@fudan.edu.cn (M.Z.); fuzhibingcn@163.com (Z.F.); wangchy807@caep.cn (C.W.); 2 Shanghai EBIT Lab, Key Laboratory of Nuclear Physics and Ion-beam Application, Institute of Modern Physics, Department of Nuclear Science and Technology, Fudan University, Shanghai 200433, China

**Keywords:** γ-irradiation method, MnO_2_/BC hybrids, high specific capacitance, cyclic stability

## Abstract

A novel facile process for fabrication of amorphous MnO_2_/bamboo charcoal monolith hybrids (MnO_2_/BC) for potential supercapacitor applications using γ-irradiation methods is described. The structural, morphological and electrochemical properties of the MnO_2_/BC hybrids have been investigated using X-ray diffraction (XRD), field emission scanning electron microscopy (FESEM), transmission electron microscopy (TEM), cyclic voltammetry (CV), galvanostatic charge/discharge (GCD) and electrochemical impedance spectroscopy (EIS) techniques. The combination of BC (electrical double layer charge) and MnO_2_ (pseudocapacitance) created a complementary effect, which enhanced the specific capacitance and good cyclic stability of the MnO_2_/BC hybrid electrodes. The MnO_2_/BC hybrids showed a higher specific capacitance (449 F g^−1^ at the constant current density of 0.5 A g^−1^ over the potential range from –0.2 V to 0.8 V), compared with BC (101 F g^−1^) in 1 M of Na_2_SO_4_ aqueous electrolyte. Furthermore, the MnO_2_/BC hybrid electrodes showed superior cycling stability with 78% capacitance retention, even after 10,000 cycles. The experimental results demonstrated that the high performance of MnO_2_/BC hybrids could be a potential electrode material for supercapacitors.

## 1. Introduction

With the rapid increase of global energy demand and the depletion risk of fossil fuels, developing alternative sustainable, affordable, efficient and clean energy has become very urgent in recent decades [1,2]. Among energy storage devices, supercapacitors (or ultracapacitors) have attracted intense interest because of their high energy density, safe operation, super-high service life and great power density [3,4,5]. Due to these features they have broad application areas, such as hybrid vehicles, pulse power systems and digital products [6,7,8]. Depending on the charge storage mechanism, supercapacitors are generally classified into electrical double layer charge (EDLC) and pseudoprocess charge storage. The former stores charge electrostatically in double layers, whereas the latter stores charge on the surface of the electrode active materials as faradaic redox reactions. In general, carbon-based materials are EDLC type [9], while transition metal oxides and conducting polymers are pseudocapacitor type materials [10].

The charge storage of pseudocapacitors is much higher in comparison with that of EDLCs. More recently, transition metal oxides, such as RuO_2_, MnO_2_, NiO, SnO_2_ and WO_3_, have been widely studied as supercapacitor electrode materials because they generate capacitances from fast superficial redox reactions [11,12,13]. Among many of the reported transition metal oxides, RuO_2_ has been extensively studied as a suitable material with excellent capacitive performance. However, RuO_2_ is expensive and rare, constraining its wide practical applications in electrode materials [14]. Except for its poor electric conductivity (10^‒5^–10^‒6^ S cm^−1^), MnO_2_ is promising as an electrode material due to its natural abundance, various morphology, high theoretical specific capacitance (~1100 F g^−1^), high density, environmental friendliness and low-cost [15,16]. However, the low electrical conductivity also leads to the decrease of its specific capacitance from theoretical values and limits its wide application. Therefore, many researchers have focused on incorporating MnO_2_ with highly conductive carbon materials to establish a hybrid type material which combines the advantages of each component [16,17,18]. Yuan et al. fabricated porous MnO_2_/rice husk derived carbon composites for supercapacitors by in situ chemical precipitation, with outstanding cycling stability (80.2% retention after 5000 cycles), and with specific capacitance of about 210.3 F g^−1^ at 0.5 A g^−1^ [6]. Li et al. also used a chemical precipitation method when designing MnO_2_ nanoflakes/hierarchical porous carbon nanocomposites with a great improvement in specific capacitance (326.9 F g^−1^ at 1 A g^−1^) [19]. Li et al. reported hovenia-acerba-like hierarchical MnO_2_/C hybrids which showed a performance of 132 F g^−1^ at 0.5 A g^−1^ [20].

Among conductive materials, activated carbon-based materials are the most promising candidates for supercapacitor applications due to their unique characteristics of large surface area, high electrochemical stability and conductivity [21,22,23,24]. In carbon materials, bamboo charcoal draws research attention for its extraordinarily porous microstructure, cost-efficiency and high absorptive capacity [22,23,25,26,27,28,29,30]. Li et al. studied water bamboo-derived porous carbon with a maximum specific capacitance of 268 F g^−1^ at a current density of 1 A g^−1^ in 6 M of KOH electrolyte and showed good capacity retention of 97.28%, even over 5000 cycles at a current density of 10 A g^−1^ [25]. Yang et al. also synthesized BC by KOH activation, and the specific capacitance retention was more than 91% after 3000 cycles [22]. Impressively, BC has long-lasting life, while MnO_2_ has high energy density. In this respect, combining each component advantage from BC and MnO_2_ to improve the performance might bring novel and excellent properties for supercapacitors. However, so far, to the best of our knowledge, nearly no works have been done on this aspect.

For decades, researchers have reported many ways to fabricate MnO_2_/carbon-based materials, such as a traditional hydrothermal method, chemical precipitation methods, electro-deposition methods, laser/microwave method and sol-gel method [30,31]. Comparing with these methods, as we previously reported [32], the radiation method is facile and fast without using a chemical reagent or even a heat process, and this γ-radiation method has been widely used in biology [33,34,35]. Moreover, we have successfully applied the radiation method for H_2_ storage [32]. However, so far nearly no studies have been conducted on the preparation of metal oxides and carbon hybrid materials for supercapacitor applications by an irradiation method. The formation mechanism for fabrication materials of irradiation methods also needs further study. In order to broaden the application of this method and further improve the capacitive performance, there is an urgent need to develop this method.

In this work, novel amorphous MnO_2_/BC hybrids were designed and fabricated by a facile γ-irradiation strategy. Morphologies and microstructures of the samples were investigated by X-ray diffraction (XRD), field emission scanning electron microscopy (FESEM) and transmission electron microscopy (TEM), while cyclic voltammetry (CV), galvanostatic charge/discharge (GCD) and electrochemical impedance spectroscopy (EIS) were carried out to study capacitive properties. The electrochemical results demonstrated that MnO_2_ hybrids delivered a high specific capacity (499 F g^−1^ at a current density of 0.5 A g^−1^) and excellent cycle stability (78% capacitance retention even after 10,000 cycles at 1 A g^−1^). The combination of bamboo (EDLC) and MnO_2_ (pseudocapacitance) provided short ion diffusion paths and rapid electronic transport, indicating good performance for supercapacitor applications.

## 2. Materials and Methods

### 2.1. Preparation of MnO_2_/BC Hybrids

**Figure 1:**
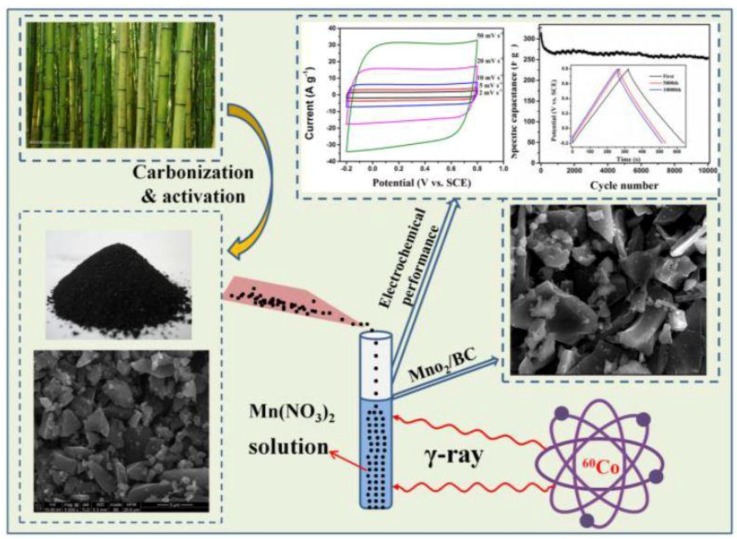
Schematic diagram for the synthesis of MnO_2_/BC hybrids and its supercapacitor performance.

All the chemical reagents and solvents in this work were analytical grade and used without additional purification. In a typical process, MnO_2_/BC hybrids were prepared as follows. Mn(NO_3_)_2_ (3 mg) was added to deionized water (20 mL) under stirring until complete dissolution for 20 min in a glass vial at room temperature. Then, the BC monoliths (0.2 g) were slowly impregnated with 10 mL of Mn(NO_3_)_2_ solution. After 30 min of continuous stirring, 2-Propanol was added with the proper amount to scavenge H* and OH* radicals which were generated during irradiation. The mixture was irradiated at room temperature with a ^60^Co γ-ray source at a dose rate of 200 Gy min^−1^, and the total dose was 500 kGy. The product was collected by centrifugation and rinsed several times with deionized (DI) water and ethanol, and then dried at 60 °C for 12 h. Figure 1 shows the schematic diagram for the synthesis of MnO_2_/BC hybrids and its supercapacitor performance.

For comparison, Bamboo charcoal monoliths were prepared by carbonization of natural bamboo with the KOH-modified method, as described elsewhere [21,26]. The bamboo was washed to remove dust, cut in to small pieces and then dried at 80 °C. After preheating under N_2_ gas at 500 °C for 3 h in a furnace, the as-prepared materials were ground to powder and mixed with KOH (KOH/C = 1:1) to further carbonize at 850 °C for 4h under a N_2_ atmosphere. The obtained materials were washed several times with 1 M HCl solution and DI water and then dried at 60 °C.

### 2.2. Materials Characterization

The crystal structure of the MnO_2_/BC hybrids was characterized by powder X-ray diffraction (XRD, X’pert PRO, PANalytical B. V, Holand) employing monochromatized Cu Kα incident radiation. The morphology and microstructure of the materials were investigated by field emission scanning electron microscopy (FESEM, Nova 600i, FEI, Hillsboro, OR, USA) equipped with energy dispersive X-ray spectroscopy (EDS) analysis operated at 15 kV and transmission electron microscopy (TEM, FEI-2100 Plus, JEOL, Japan). The surface chemical composition and oxidation state of the MnO_2_/BC hybrids were determined using X-ray photoelectron spectroscopy (XPS, Kratos Analytical Ltd., Manchester, UK). The nitrogen adsorption/desorption isotherms were carried out at 77 K using a Quantachrome Autosorb-1 instrument (Quantachrome Corporation, Florida, USA). The specific surface area and pore size distribution were calculated by the Brunauer-Emmett-Teller (BET) and density functional theory (DFT) methods, respectively.

### 2.3. Electrochemical Measurements

The electrochemical tests were carried out at room temperature in a conventional three- and two-electrode configuration, with 1 M of Na_2_SO_4_ as an electrolyte using an electrochemical workstation (CHI 660E, Chenhua, Shanghai, China), MnO_2_/BC as a working electrode, platinum foil as a counter electrode and a saturated calomel electrode (SCE) as a reference electrode. For electrochemical tests, the MnO_2_/BC (80%) was mixed with acetylene black (10%) and a binder (polyvinylidene fluoride, PVDF, 10%) in Nmethyl-2-pyrrolidone (NMP) to form slurry. Then, the slurry was coated on glassy carbon to fabricate a working electrode with the mass loading of the active material at about 1 mg. The potential range for CV tests was from 0.2 V to 0.8 V, and the measurement range for EIS tests was between 0.01 Hz and 100 kHz, with an alternating current amplitude of 5 mV.

## 3. Results and Discussion

### 3.1. Characterization of MnO_2_/BC Hybrids

**Figure 2:**
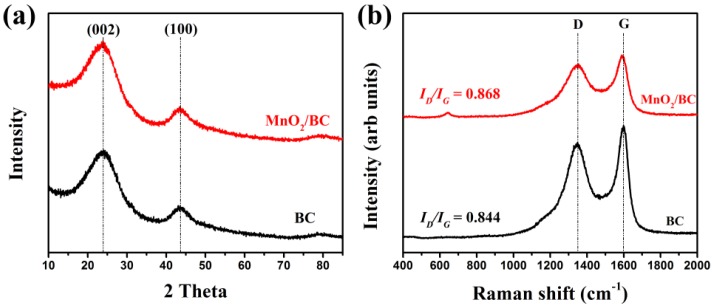
(**a**) XRD pattern and (**b**) Raman spectrum of the BC and MnO_2_/BC hybrids.

Crystal structures of the as-prepared BC and MnO_2_/BC hybrids were first studied by XRD, and their XRD patterns were shown in Figure 2a. In the XRD pattern of the BC, just like any other pyrolytic carbons, two broad peaks near 23° and 43°correspond to the graphite reflections from (002) and (100) crystal planes respectively, which can be identified as the amorphous forms of BC [6,36]. For the MnO_2_/BC hybrids, two broad peaks of BC also appeared and almost no obvious difference between BC and MnO_2_/BC hybrids could be found. Owing to the low content or the amorphous forms of MnO_2_, no clear diffraction peaks for MnO_2_ can be detected [37,38]. Ultimately, no other impurity phase peak is found from these patterns, indicating the high purity of the products. A Raman spectrometer was used to further investigate the structural features of the BC and MnO_2_/BC hybrids, as shown in Figure 2b. We can see two remarkable peaks for both BC and MnO_2_/BC hybrids at 1353 and 1591 cm^−1^, which are respectively assigned to the typical D band from the presence of an sp3 defect, and the G band from the in-plane vibration of sp2 carbon atoms of BC [39,40]. Furthermore, the *I*_D_/*I*_G_ ratio is widely applied in evaluating graphitization degree and structural defects. Compared with BC, it can be observed that the *I*_D_/*I*_G_ value for MnO_2_/BC hybrids ascends from 0.844 to 0.868, manifesting a slight increase in the defect ratio of the deposition of MnO_2_ and a negligible effect on the structure of the BC. The peak of MnO_2_/BC hybrids were located at the low wave number region of 640 cm^−1^, which matches well with the major feature of Mn–O symmetric stretching vibration, around 635–650 cm^−1^ for MnO_2_ [40,41,42,43]. This result is in good agreement with the previous reports, providing evidence for the formation of amorphous MnO_2_ in BC. Thus, XRD and Raman results can clearly demonstrate the successful preparation of the MnO_2_/BC hybrids. The presence of MnO_2_/BC hybrids can be confirmed in depth by the results of SEM, TEM and XPS.

**Figure 3:**
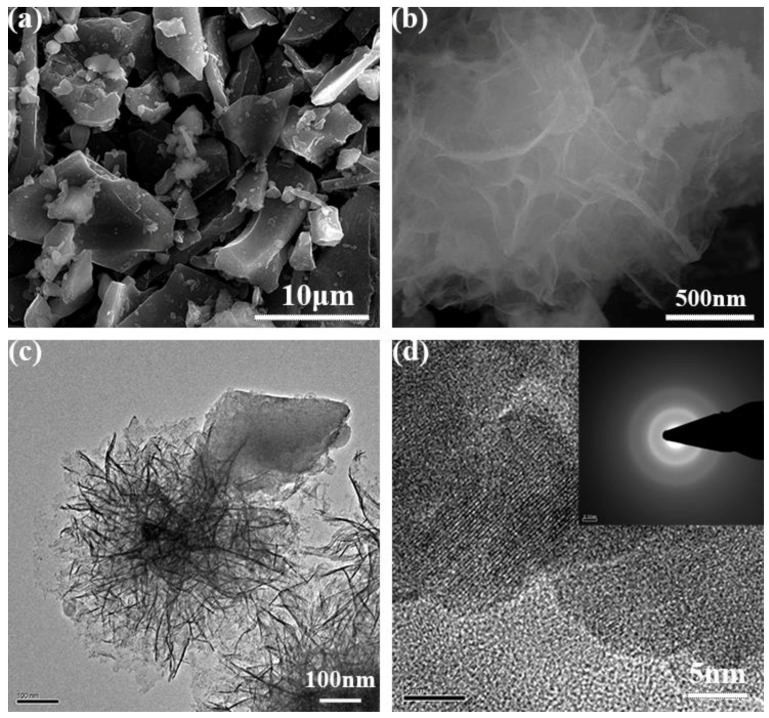
(**a**,**b**) Typical SEM images of the MnO_2_/BC hybrids. (**c**,**d**) TEM and HRTEM images of the MnO_2_/BC hybrids.

The surface morphologies of BC and MnO_2_/BC hybrids were characterized by SEM and TEM. As shown in Figure S1, we can see clearly that the BC has a smooth surface and irregular forms. Figure 3a shows the morphology of MnO_2_/BC hybrids, and the skeleton of BC can be seen clearly with a random distribution of MnO_2_. In particular, the amorphous MnO_2_ possesses a flower-like structure (Figure 3b). EDS mapping was further used to demonstrate the formation of the MnO_2_/BC hybrids, and is shown in Figure S2. Obviously, the C, Mn and O elements exist in the MnO_2_/BC hybrids. To investigate more detailed information about the microstructural features, TEM and high-resolution TEM micrographs of MnO_2_/BC hybrids are shown in Figure 3c,d. In the TEM image of MnO_2_/BC hybrids, it is clearly revealed that the amorphous MnO_2_ is successfully connected with BC. Figure 3d shows an HRTEM image of MnO_2_/BC and the inset refers to the corresponding selected area electron diffraction (SAED) pattern, further confirming the amorphous structure of MnO_2_. Therefore, as confirmed by the above SEM, TEM and EDS elemental mapping results, the amorphous MnO_2_/BC hybrids have been successfully synthesized via a simple γ-irradiation method. This great interfacial contact between MnO_2_ and BC is favorable for the electronic transport process, thus resulting in enhanced electrochemical performance [44].

**Figure 4:**
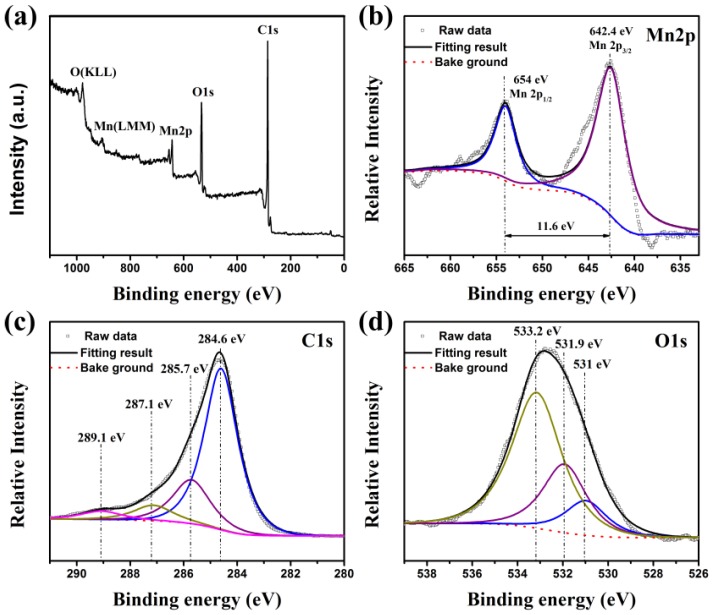
The XPS spectra of MnO_2_/BC hybrids: (**a**) Survey scan spectrum; (**b**) W 2p core level spectrum; (**c**) C 1s core level spectrum; (**d**) O 1s core level spectrum.

The detailed surface chemical composition and valence state of the MnO_2_/BC hybrids were probed by XPS measurements. Figure 4a shows the XPS spectrum of the MnO_2_/BC hybrids, and the full survey scan spectrum shows that only three elements (Mn, O and C) are contained in the sample, confirming the high purity of the MnO_2_/BC hybrids. In the high-resolution scan, Figure 4b shows the XPS spectrum of the Mn 2p doublet peak. The peaks were located at binding energies of 642.4 and 654 eV, which correspond to the Mn 2p_3/2_ and Mn 2p_1/2_, respectively. The observed energy position of the doublet is in good agreement with the literature for the Mn^4^^+^ oxidation state [45,46]. The energy separation between Mn 2p_3/2_ and Mn 2p_1/2_ is 11.6 eV, indicating a normal state of Mn^4^^+^ in MnO_2_ [3,47]. Therefore, MnO_2_ successfully grows on BC to form the MnO_2_/BC hybrids, which is a good analogue of the Raman and EDS studies. The XPS spectrum of C 1s from the MnO_2_/BC hybrids (seen in Figure 4c) is also decomposed into four peaks at 284.6, 285.7, 287.1 and 289.1 eV, which are attributed to (C–C/C=C sp^2^), (C–O), (C=O) and (–COOH) [48,49], respectively, suggesting bonding between carbon atoms of BC with oxygen atoms of MnO_2_ facilitates the charge transportation and conductivity of the hybrids [14,50]. As shown in Figure 4d, the O 1s spectrum can be resolved into the three binding energy components of 531, 531.9 and 533.2 eV, which are attributed to the Mn–O–Mn bond of the tetravalent oxide, Mn–O–H bond of hydroxide and H–O–H bond of water, respectively [49,51].

**Figure 5:**
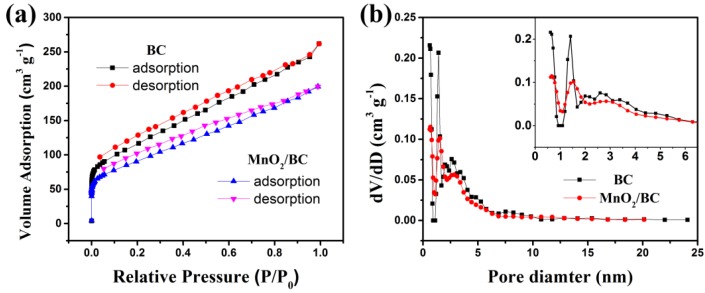
(**a**) N_2_ adsorption desorption isotherms and (**b**) pore size distribution of MnO_2_/BC hybrids.

As we know, the specific surface area of the electrode materials is an important factor for supercapacitor performance. The nitrogen adsorption/desorption isotherms and corresponding DFT pore size distribution curves of BC and MnO_2_/BC hybrids are shown in Figure 5. The detailed porous properties of BC and MnO_2_/BC hybrids are summarized in Table 1. Both BC and MnO_2_/BC hybrids (Figure 5a) show type I and IV isotherm curves with type H4 hysteresis loops (IUPAC) [42], indicating micropores and mesopores exist in the samples. The high relative pressure also refers to the macroporous nature of the BC and MnO_2_/BC hybrids. Detailed DFT pore size distribution structure is shown in Figure 5b. Notably, the BET surface area of BC and MnO_2_/BC hybrids are 414 and 323 m^2^ g^−1^, respectively (Table 1). The BET surface area of the MnO_2_/BC hybrids is smaller than that of the BC, indicating the amorphous MnO_2_ partially blocked the pores of the BC, and this phenomenon is consistent with previous reports [52,53]. In addition, the proper pore size distribution and high specific surface area of MnO_2_/BC hybrids are of huge benefit for faster charge transfers and suitable paths for the electrolyte ions, resulting in excellent electrochemical performance [54,55,56,57].

### 3.2. Electrochemical Performance of MnO_2_/BC Hybrids

**Figure 6:**
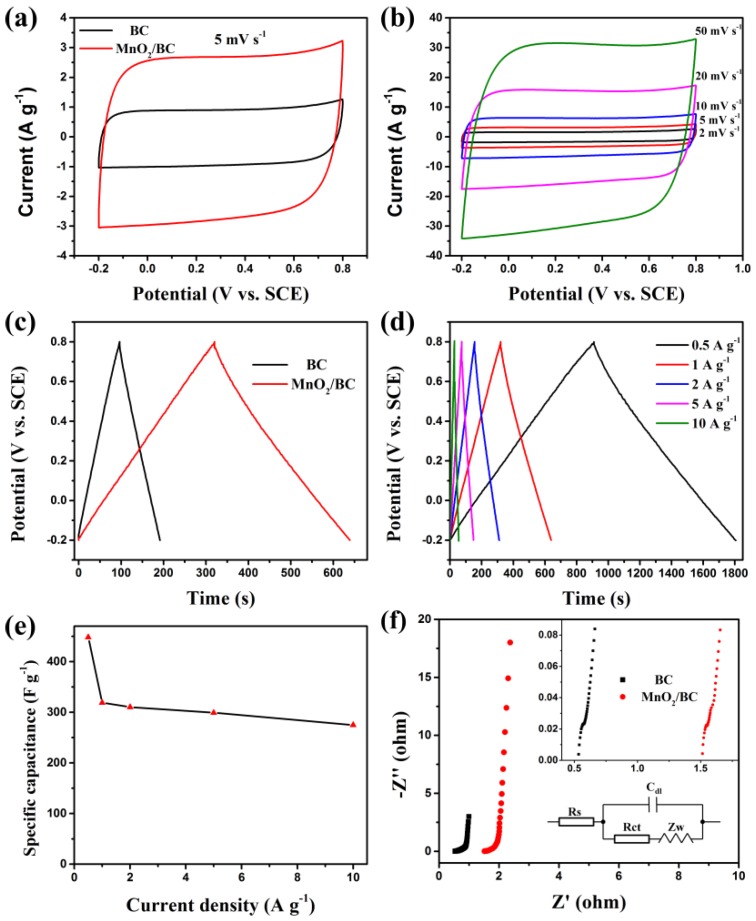
(**a**) The CV curves of BC and MnO_2_/BC electrodes at a scan rate of 5 mV s^−1^ in 1 M of Na_2_SO_4_, (**b**) the CV curves of the MnO_2_/BC electrode at different scan rates, (**c**) GCD curves of BC and MnO_2_/BC electrodes at a current density of 1 A g^−1^, (**d**) GCD curves of the MnO_2_/BC electrode at different current densities, (**e**) specific capacitance of MnO_2_/BC electrode at different current density, (**f**) EIS of the BC and MnO_2_/BC electrode (The inset shows the expanded high-frequency region of Nyquist plots).

Cyclic voltammetry (CV) is a powerful technique for the determination of potentials involved. Figure 6a shows the CV curves of the BC and MnO_2_/BC electrodes at the same scan rate of 5 mV s^−1^ with a potential window between −0.2 V to 0.8 V in 1 M of Na_2_SO_4_ electrolyte. Compared with BC, having almost no obvious redox peaks in CV curves of MnO_2_/BC is a very common feature among various MnO_2_ analogues [58,59]. Moreover, it is well known that the charge which is stored within the capacitor may be determined by integrating the CV. The observed integrated area and the current density of the CV curve for the MnO_2_/BC electrode are much higher than those for the BC electrode, indicating that the contribution of MnO_2_ to the specific capacitance of MnO_2_/BC and the complementary effects of MnO_2_ and BC are significant [59]. The results obtained here are also consistent with the SEM, TEM, XPS and N_2_ adsorption desorption tests, suggesting the MnO_2_/BC hybrid morphology provides good contact enabling a fast charge intercalation/deintercalation process. Figure 6b shows the CV curves of the MnO_2_/BC in 1 M Na_2_SO_4_ at different scan rates (2, 5, 10, 20 and 50 mV s^−1^) over a potential window of −0.2 V to 0.8 V. The corresponding CV curves of BC are provided in Figure S3a. The increase in area under the curve with scan rate is clearly observed, indicating an excellent capacitance behavior and high-rate capability of the electrode.

Charge–discharge measurements were conducted under galvanostatic conditions at different applied current densities. The GCD plots of the BC and MnO_2_/BC electrodes at a current density of 1 A g^−1^ are presented in Figure 6c. According to the galvanostatic discharge curves, the specific capacitance C_s_ (F g^−1^) of the electrode is calculated based on the following equation:

$$C_{s}\left(Fg^{-1}\right)=\frac{I\Delta t}{m\Delta V}$$

where *I* (mA) is the current used for charge-discharge, Δ*t* (s) is the time of the galvanostatic discharging, Δ*V* (V) is the potential drop during discharge, and *m* (mg) is the weight of the active material in the electrode. Additionally, the discharge time of MnO_2_/BC is much longer than BC, showing higher capacitance. This result is in consistent with the CV tests. GCD curves of MnO_2_/BC and BC (Figure S3b) electrodes recorded at 0.5, 1, 2, 5 and 10 A g^−1^ are shown in Figure 6d. With the increasing charge and discharge currents, the highly linear and nearly symmetric relationship between potential versus time was also observed, suggesting the desired fast charge and discharge property of the materials. No obvious internal resistance (IR) drop of the BC (Figure S3b) and MnO_2_/BC electrode was observed for any of the curves, which indicates high conductivity of the materials. The higher C of MnO_2_/BC is due to the achievement of optimal complementary effects between MnO_2_ and BC [18]. As shown in Figure 6e, the plot of calculated Cs of MnO_2_/BC is 449, 319, 310, 299 and 275 F g^−1^ at current densities of 0.5, 1, 2, 5 and 10 A g^−1^, respectively, demonstrating that the specific capacitance decreases with increasing current density [60]. Furthermore, about 61% of the capacitance was retained when the current density increased from 0.5 to 10 A g^−1^. These results are comparable to those MnO_2_-carbon based electrodes in earlier reports (Table S1). In summary, the enhancement in the electrochemical performance of the MnO_2_/BC hybrids is mainly explained as follows: (i) The great interfacial contact between MnO_2_/BC and BC provides short ion diffusion paths and rapid electronic transports; (ii) the complementary effects of the hybrids are favorable for charge storage by EDLC and pseudocapacitance.

The EIS study was conducted to elucidate electrical conductivity and ion transfer features of BC and MnO_2_/BC electrodes. Electrochemical impedance characteristics of the electrodes were investigated in a frequency range from 100 kHz to 0.01 Hz with an alternating current amplitude of 5 mV in 1 M of Na_2_SO_4_ electrolyte. Figure 6f displays the typical Nyquist plots of these BC and MnO_2_/BC samples and the inset figure shows the magnified plots. All the EIS curves show a semicircle in the high-frequency and a straight line in the low frequency regions. According to previous reports [53,61,62], a straight line should relate to the diffusion of electrolyte ions into the active electrode, whereas the semicircle can be assigned to the charge transfer resistance (Rct) due to the faradic reactions and double-layer capacitance of the electrode/electrolyte interface. The intercept of the semicircle with real axis gives Rs, which is the sum of the ionic resistance of electrolyte, the intrinsic resistance of the active materials and the contact resistance at the interface between electrode and electrolyte, with the Rs values of BC and MnO_2_/BC hybrids of 0.57 and 1.51 Ω, respectively. In addition, comparing with the BC electrode, the MnO_2_/BC electrode exhibits much larger Rct, Rs and electrolyte ion diffusion, which is mainly due to the poor electric conductivity of MnO_2_, partially blocked pores of the BC and the decrease of BET specific surface area. These results coincide with other reports [20,39].

**Figure 7:**
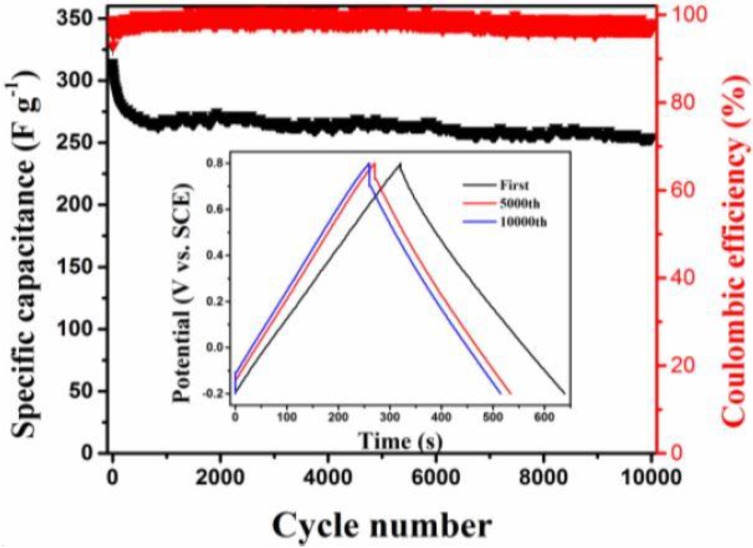
Cycling stability of the MnO_2_/BC electrode at a current density of 1 A g^−1^ in 1 M of Na_2_SO_4_ electrolyte (the inset shows GCD curves at different cycles).

Long term cycling stability is the key factor for evaluating the practical applications of electrodes. In order to explore this, the cycling stability of MnO_2_/BC hybrids was further investigated by repeating the constant current charge/discharge test between −0.2 V to 0.8 V at a current density of 1 A g^−1^ for 10,000 cycles in 1 M of Na_2_SO_4_ electrolyte as shown in Figure 7. Impressively, after 10,000 continuous charge/discharge cycles, it is observed that the initial capacitance (328 F g^−1^) of MnO_2_/BC electrode slightly decreased to 255 F g^−1^ and about 78% of the initial capacitance was retained, possibly owing to the desquamation of MnO_2_ from BC monoliths [6]. The inset shows the 1st, 5000th and 10,000th GCD curves, respectively, indicating that the charging/discharging profiles retain excellent linearity and symmetry even after 10,000 cycles. The coulombic efficiency of MnO_2_/BC hybrids is approximately 100%. Comparing with other studies, to the best of our knowledge, the MnO_2_/BC hybrid electrode shows comparable cycling stability (Table S1). Moreover, the BC used in our work is much cheaper, and the radiation method is facile and fast. Ragone plots tested by two symmetric electrode configurations of the MnO_2_/BC hybrids compared with the values of similar MnO_2_ based supercapacitors are shown in Figure S4. The highest energy density is 23 Wh kg^−1^ at 50 kW kg^−1^. Therefore, the complementary effect of MnO_2_ and BC plays an important role in the sufficient diffusion of electrolyte ions. This result confirms that the prepared MnO_2_/BC hybrids were highly stable as a novel supercapacitor electrode.

## 4. Conclusions

In summary, we successfully synthesized MnO_2_/BC hybrids via a facile γ-irradiation method. The electrochemical capacitive behaviors of the MnO_2_/BC hybrids and BC have been demonstrated in 1 M Na_2_SO_4_ electrolyte between −0.2 V to 0.8 V. In comparison with the BC electrode, MnO_2_/BC hybrid electrodes showed a higher specific capacitance (449 F g^−1^ at 0.5 A g^−1^) and superior cycling performance (about 78% retention even after 10,000 cycles). The excellent performance achieved by the MnO_2_/BC hybrid electrodes could be ascribed to the complementary effect of conductive BC and the pseudocapacitive behavior of MnO_2_. We hope that this γ-irradiation method would also pave a new way for the design of other conducting semiconductors as promising electrode materials with enhanced performance for energy storage device applications, as well as expanding practical application fields, such as selective adsorption and catalyst support.

## Figures and Tables

**Table 1:** Relevant parameters for the pore properties determined by nitrogen adsorption and desorption isotherms of the BC and MnO_2_/BC hybrids.

Sample	BET Specific Surface Area/ (m^2^ g^−1^)	Average Pore Size/ (nm)	Total Pore Volume/ (cm^3^ g^−1^)
BC	414	3.91	0.404
MnO_2_/BC hybrids	323	3.82	0.309

## Supplementary Materials

The following are available online at http://www.mdpi.com/2079-4991/8/7/533/s1, Figure S1: (a) Typical SEM images of BC. (b) HRTEM images of the BC (the inset shows SAED pattern), Figure S2: The corresponding elemental mapping of C, Mn, and O for the MnO_2_/BC hybrids), Figure S3: (a) The CV curves of the BC electrode at different scan rates, and (b) GCD curves of the BC electrode at different current densities, Figure S4: Ragone plots of the MnO_2_/BC hybrids compared with the values of similar MnO_2_ based supercapacitors, Table S1: Comparison of various MnO_2_-carbon based electrodes in recent years as supercapacitors, Table S2: Impedance parameters derived by equivalent circuit model for BC and MnO_2_/BC electrodes.
