# Supplementary material for: A Novel Radiation Method for Preparing MnO2/BC Monolith Hybrids with Outstanding Supercapacitance Performance
Source: Nanomaterials (Basel). 2018 Jul 14;8(7):533. doi: 10.3390/nano8070533 (PMC6070953; doi:10.3390/nano8070533)
Supplement: Supplementary file 1 [file nanomaterials-08-00533-s001.pdf]

Supplemental Materials

A Novel Radiation Method for Preparing MnO_2/BC Monolith Hybrids with Outstanding Supercapacitance Performance

Fan Yang [1†], Xichuan Liu [1,2†], Rui Mi [1], Lei Yuan [1], Xi Yang [1], Minglong Zhong [1], Zhibing Fu [1], Chaoyang Wang [1], Yongjian Tang [1,*]

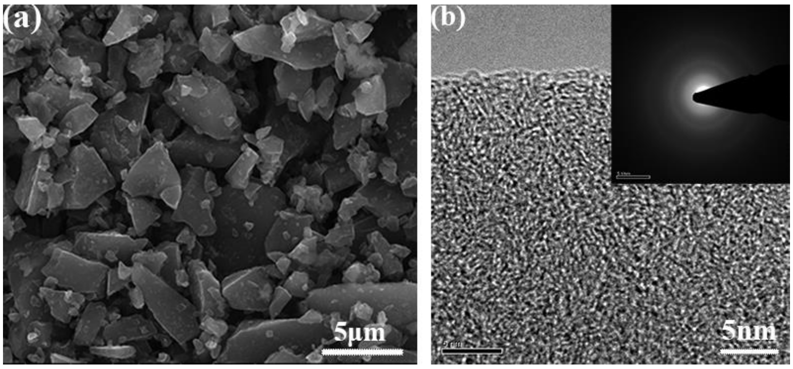

Figure S1. (**a**) Typical SEM images of BC, (**b**) HRTEM images of the BC (the inset shows SAED pattern).

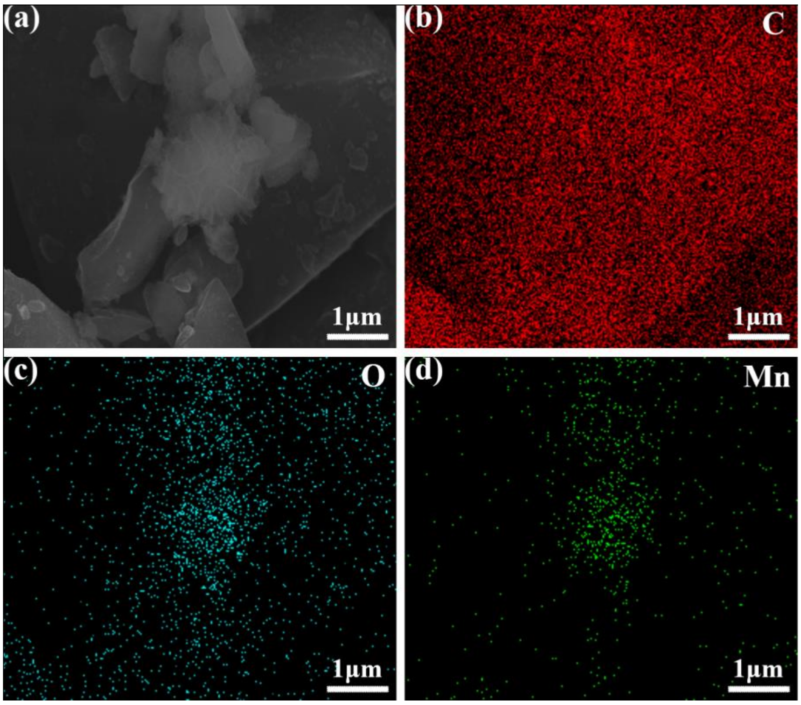

Figure S2. EDS mapping results of C, Mn, and O for the MnO_2/BC hybrids.

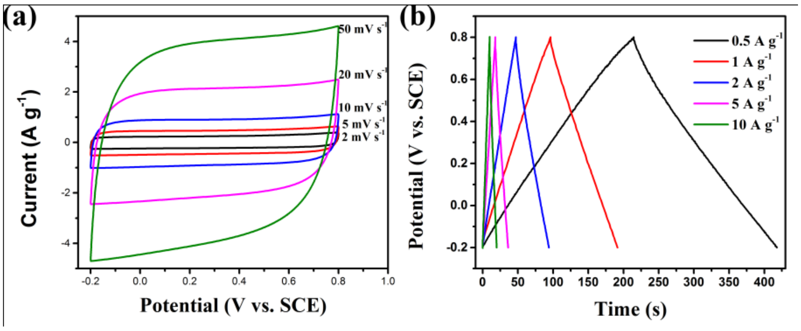

Figure S3. (**a**) The CV curves of the BC electrode at different scan rates and (**b**) GCD curves of the BC electrode at different current densities.

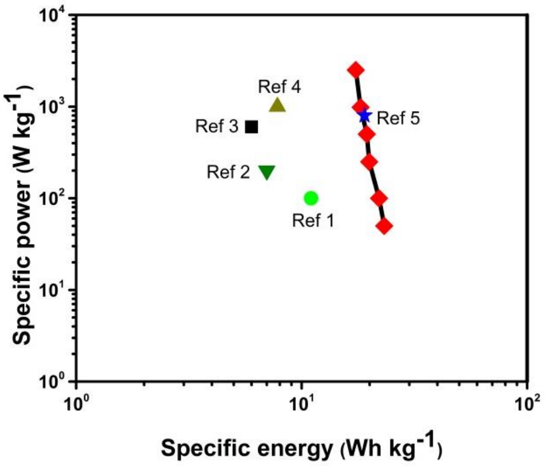

Figure S4. Ragone plots of the MnO_2/BC hybrids compared with the values of similar MnO_2 based supercapacitors.

Table S1. Comparison of various MnO_2-carbon based electrodes used in recent years as supercapacitors.

Sr. No.	Material	Synthesis method	Two/Three electrode	Specific capacitance	Cycling Stability	Ref.
1	MnO_2/carbon	chemical precipitation	Three electrode	132 F g^{-1} at 0.5 A g^{-1}	1000 (100%)	[6]
2	MnO_2/HPCs	chemical precipitation	Three electrode	326.9 F g^{-1} at 1 A g^{-1}	1000 (100%)	[7]
3	MnO_2/RHCs	co-precipitation	Three electrode	210.3F g^{-1} at 0.5 A g^{-1}	5000 (80.2%)	[8]
4	MnO_2/carbon nanotube	electrodeposition	Three electrode	300 F g^{-1} at 1 A g^{-1}	1000 (38.4%)	[9]
5	MnO_2/carbon nanofiber	Electrospinning method	Two electrode	228 F g^{-1} at 50 mV s^{-1}	1000 (88%)	[10]
6	CNFs/ MnO_2	Electrospinning method	Three electrode	151.1 F g^{-1} at 1 A g^{-1}	8000 (90%)	[11]
7	MnO_2/3D porous carbon	Hydrothermal method	Three electrode	386 F g^{-1} at 1 A g^{-1}	5000 (83%)	[12]
8	MnO_2/BC	γ-irradiation method	Three electrode	449 F g^{-1} at 0.5 A g^{-1}	10,000 (78%)	This work

Table S2. Impedance parameters derived by equivalent circuit model for BC and MnO_2/BC electrodes.

Sample	Rs (Ω)	Rct (Ω)
BC	0.57	0.11
MnO_2/BC	1.51	0.19

1. Nilesh R. Chodankar, S.-H.J., Do-heyong Kim. Low-cost superior symmetric solid-state supercapacitors based on mwcnts/MnO_2 nanocomposite thin fil. *J. Taiwan Inst. Chem. Eng.* **2017**, *80*, 503-510.
2. Wen, Y.; Qin, T.; Wang, Z.; Jiang, X.; Peng, S.; Zhang, J.; Hou, J.; Huang, F.; He, D.; Cao, G. Self-supported binder-free carbon fibers/MnO_2 electrodes derived from disposable bamboo chopsticks for high-performance supercapacitors. *J. Alloys Compd.* **2017**, *699*, 126-135.
3. Jia-Wei Wang, Y.C., Bai-Zhen Chen. Synthesis and control of high-performance MnO_2/carbon nanotubes nanocomposites for supercapacitors. *J. Alloys Compd.* **2016**, *688*, 184-197.
4. He, Y.; Du, S.; Li, H.; Cheng, Q.; Pavlinek, V.; Saha, P. MnO_2/polyaniline hybrid nanostructures on carbon cloth for supercapacitor electrodes. *J. Solid State Electrochem.* **2016**, *20*, 1459-1467.
5. He, Y.; Chen, W.; Li, X.; Zhang, Z.; Fu, J.; Zhao, C.; Xie, E. Freestanding three-dimensional graphene/MnO_2 composite networks as ultralight and flexible supercapacitor electrodes. *Acs Nano* **2013**, *7*, 174.
6. Qun Li, J.H., Dequan Liu, Hongwei Yue, Shuai Bai, Boli Liu, Lili Gu, Deyan He. Facile preparation of hovenia-acerba-like hierarchical MnO_2/C composites and their excellent energy storage performance for supercapacitors. *J. Alloys Compd.* **2017**, *693*, 970-978.
7. Li, H.; Jiang, L.; Cheng, Q.; He, Y.; Pavlinek, V.; Saha, P.; Li, C. MnO_2 nanoflakes/hierarchical porous carbon nanocomposites for high-performance supercapacitor electrodes. *Electrochim. Acta* **2015**, *164*, 252-259.
8. Chuanjun Yuan, H.L., Haiyan Lua, Endong Xing, Yusi Zhang, Bingyao Xie. Synthesis of hierarchically porous MnO_2/rice husks derived carbon composite as high-performance electrode material for supercapacitors. *Appl. Energy* **2016**, *178*, 260-268.
9. Tagsin, P.; Klangtakai, P.; Harnchana, V.; Amornkitbamrung, V.; Pimanpang, S.; Kumnorkaew, P. Enhanced specific capacitance of an electrophoretic deposited MnO_2-carbon nanotube supercapacitor. *Journal of the Korean Physical Society* **2017**, *71*, 997-1005.
10. Lee, D.G.; Kim, J.H.; Kim, B.-H. Hierarchical porous MnO_2/carbon nanofiber composites with hollow cores for high-performance supercapacitor electrodes: Effect of poly(methyl methacrylate) concentration. *Electrochim. Acta* **2016**, *200*, 174-181.
11. Peigong Ning, X.D., Xiaokang Ju, Xiaoping Lin, Xiaobin Tong, Xi Pan,; Taihong Wang, Q.L. Facile synthesis of carbon nanofibers/MnO_2 nanosheets as high-performance electrodes for asymmetric supercapacitors. *Electrochim. Acta* **2016**, *210*, 754-761.
12. Luo, X.; Yang, J.; Yan, D.; Wang, W.; Wu, X.; Zhu, Z. MnO_2-decorated 3d porous carbon skeleton derived from mollusc shell for high-performance supercapacitor. *J. Alloys Compd.* **2017**, *723*, 505-511.
